# Supplementary material for: The genomic landscape of polymorphic human nuclear mitochondrial insertions
Source: Nucleic Acids Res. 2014 Oct 27;42(20):12640–9. doi: 10.1093/nar/gku1038 (PMC4227756; doi:10.1093/nar/gku1038)
Supplement: SUPPLEMENTARY DATA [file supp_42_20_12640__index.html]

The genomic landscape of polymorphic human nuclear mitochondrial insertions — The genomic landscape of polymorphic human nuclear mitochondrial insertions — SUPPLEMENTARY DATA 

# The genomic landscape of polymorphic human nuclear mitochondrial insertions

## SUPPLEMENTARY DATA

**Files in this Data Supplement:**

- SUPPLEMENTARY DATA
- SUPPLEMENTARY DATA
- SUPPLEMENTARY DATA
