# Supplementary material for: The genomic landscape of polymorphic human nuclear mitochondrial insertions
Source: Nucleic Acids Res. 2014 Oct 27;42(20):12640–9. doi: 10.1093/nar/gku1038 (PMC4227756; doi:10.1093/nar/gku1038)
Supplement: SUPPLEMENTARY DATA [file supp_gku1038_nar-02529-h-2014-File007.docx]

**Figure S1:** Number of NumtS discovered as a function of sequence coverage for each individual sample. There is a statistically significant but modest difference in the magnitude of discovered NumtS across the different coverage levels (r^2^=0.12).


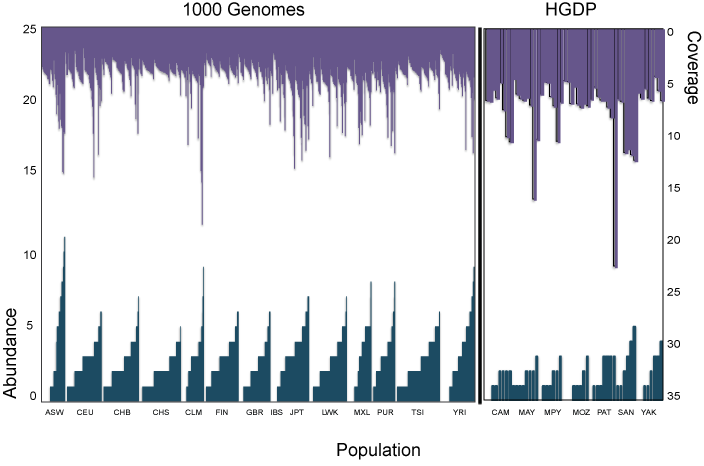


**Figure S2:** Number of NumtS discovered (bottom) and corresponding depth of sequence coverage (top) in each sample used in this analysis. While the sequencing coverage is higher on average in the HGDP samples relative to the 1000 Genomes Project samples, the mean number of NumtS identified in each sample remains essentially the same.


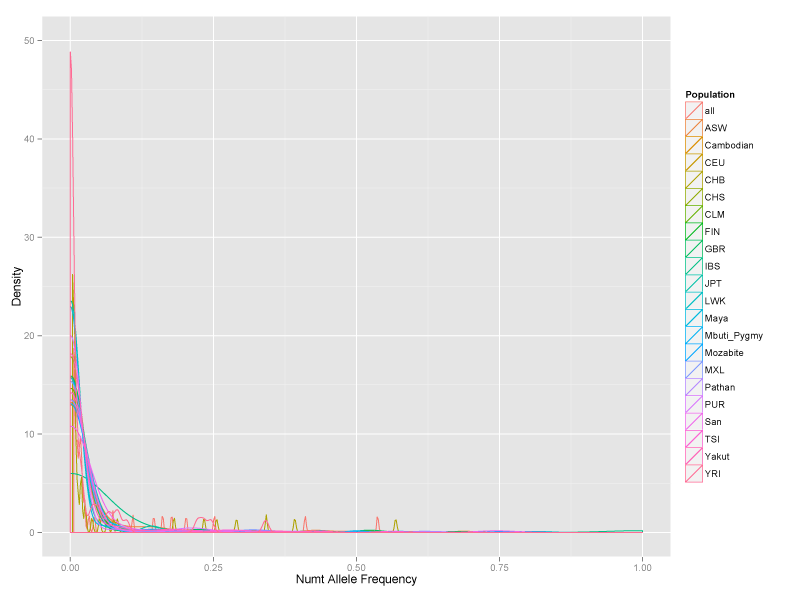


**Figure S3:** Allele frequency spectrum for Numt genotypes, stratified by population. Plot is truncated at 50 to better view lower density ranges.


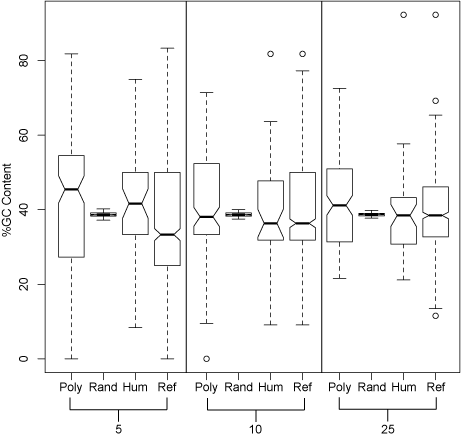


**Figure S4:** Comparison of %GC in different flanking sizes (5, 10, 25bp) of each polymorphic Numt insertion site (Poly, n=138), relative to 1000 random insertion sites throughout the genome (Rand, n=138), previously reported NumtS specific to humans (Hum, n=45) (Lang et al, 2012), and reference NumtS used in other analyses (Ref, n=610) (Tsuji et al, 2012).


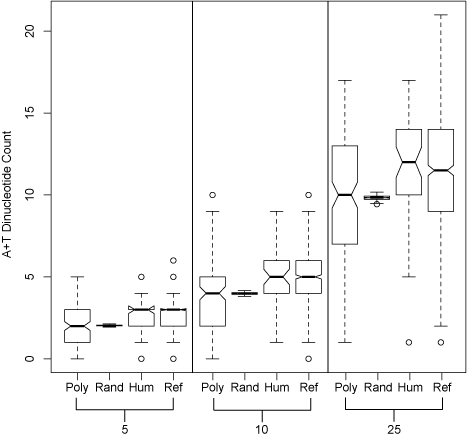


**Figure S5:** Comparison of A+T dinucleotides (AA, AT, TA, and TT) in different flanking sizes (5, 10, 25bp) of each polymorphic Numt insertion site (Poly, n=138), relative to 1000 random insertion sites throughout the genome (Rand, n=138), previously reported NumtS specific to humans (Hum, n=45) (Lang et al, 2012), and reference NumtS used in other analyses (Ref, n=610) (Tsuji et al, 2012).


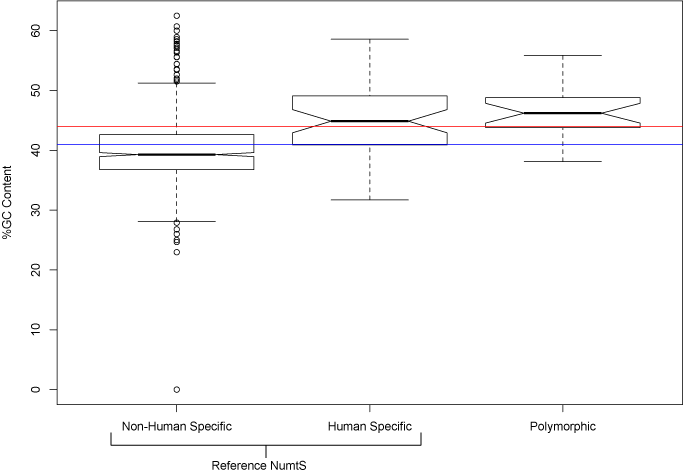


**Figure S6:** Comparison of %GC of Numt sequences between human and non-human specific reference NumtS (Lang et al, 2012) and polymorphic NumtS reported in this study. Average %GC is indicated for the reference mitochondria (red) and nuclear (blue) genomes.
